# Patient Centricity—An Empirical Research on Titanium Dental Implants and Their Adverse Effects on Health Condition

**DOI:** 10.3390/healthcare12222207

**Published:** 2024-11-05

**Authors:** Mădălin Dorel Țap, Florentina Cornelia Bîcleşanu, Octavia-Sorina Honțaru, Anamaria-Cătălina Radu

**Affiliations:** 1 Faculty of Dental Medicine, “Titu Maiorescu” University, 67 A, Str. Gheorghe Petraşcu, District 3, 031593 Bucharest, Romania; tapmadalin.dr@gmail.com (M.D.Ț.); corneliabicle@yahoo.com (F.C.B.); 2 Faculty of Sciences, Physical Education and Informatics, Department of Public Health Argeș, National University of Science and Technology Politehnica Bucharest, Pitești University Center, 1, Târgul din Vale, 110040 Pitești, Romania; sorina.hontaru@upb.ro; 3 Institute of National Economy, Romanian Academy, 13, Calea 13 Septembrie, District 5, 050711 Bucharest, Romania

**Keywords:** oral health, patient perception, oral implantology, titanium dental implant, adverse effects

## Abstract

Background/Objectives: Titanium dental implants are considered to be the most modern and effective solution for replacing lost teeth. These medical devices not only restore the chewing and aesthetic functionality of the smile but also provide a stable support for crowns, bridges or dentures. The aim of this study was to identify the perceptions of patients about titanium dental implants and their effects on the human body. Methods: A structural equation model (SEM) was conducted to study how a series of independent variables have the ability to influence the perception and intention of the patients regarding these medical devices. A data analysis was performed using WarpPLS 8.0 software. This research was conducted on a sample of 162 respondents. Results: The results illustrated that patients’ perception of titanium dental implants is explained by 71% of the independent variables analysed, and their intention to have a new implant in the next period is explained by 61%. The proposed econometric model was validated, with seven hypotheses accepted out of nine. Conclusions: Although titanium has long been used in implant dentistry, in recent years, experts have identified a number of adverse effects that can arise from its use. This study has added value both at the practical and theoretical level. Perception is influenced by respondents’ perceived advantages, by the problems perceived by the respondents regarding titanium dental implants, by the degree of awareness of the risks of titanium dental implants, and by the adverse effects experienced by respondents regarding titanium dental implants.

## 1. Introduction

An ageing population and poor oral hygiene has led to the increasing use of dental implants. They aim is to replace lost or damaged teeth by trauma. Over time, this technique has contributed to an increase in the quality of life but also in patient satisfaction [1]. In recent years, there has been an improvement in the work carried out in the field of dental implant dentistry, mostly due to both the development of new techniques and materials and also new procedures aimed at increasing the re fliability of implant therapy [2].

Titanium is one of the most widely used materials in the production of dental implants due to its unique properties [3,4]. It is seen as an extremely strong and durable metal, an aspect that gives it impressive longevity compared to other materials. It has been used for many years in oral implant dentistry [5]. Titanium dental implants were first mentioned in the literature by Per-Ingvar Branemark [6], an orthopaedic surgeon who in 1969 studied the interosseous anchorage of dentures in animals.

Subsequently, the first titanium dentures appeared in 1977 [7]. Since that time, titanium dental implants started to be increasingly used by surgeons to treat a number of problems caused by the loss of natural teeth [8,9]. They have been seen to have high market relevance, revolutionizing the field of dentistry while helping to restore oral function and facial aesthetics [10]. Specialists who have studied this field of activity have stated that one of the most important advantages of titanium refers to its biocompatibility [11]. Moreover, they considered that this material does not have the capacity to cause allergic reactions, being well tolerated by human tissues. In addition, it was observed that titanium has the ability to form a direct bond with bone through an osseointegration process [12,13], thus ensuring the stability and functionality of the dental implant.

The fairly low failure rate of titanium dental implants [14], high success rate [15], high survival rate [16] and the durability of titanium dental implants [17] are just some of the reasons why patients prefer medical devices made of titanium over other materials. All these qualities of titanium have determined the dentists to choose dental medical devices made from this material, thus providing patients with durable and effective solutions to replace lost teeth. Even though, currently, titanium is considered to be a material that has had the ability to revolutionize dental medicine by providing durable and effective solutions for replacing lost teeth, it has been observed that there are, however, a number of adverse effects as well as risks that may arise from its use [18].

The first adverse effect refers to the occurrence of adverse reactions and also sensitivity to titanium [19]. Thus, even if it is not common, titanium allergies can occur [20]. Patients may show allergic reactions [21] to titanium dental implants, experiencing symptoms such as inflammation and pain and, in some cases, even rejection of the dental implant may occur. To identify sensitivity to titanium [22], a series of tests [23,24] should be performed to confirm the patient’s allergy to this material. Implant corrosion [25] and the release of titanium ions [26] are another adverse effect that can be encountered in dental medical devices made of this material. Corrosion [27] is one of the most important factors that has the capacity to influence the biocompatibility of a dental implant [28]. Although titanium is appreciated for its resistance to corrosion, in a complex environment such as the oral cavity, corrosion processes can occur [29,30].

The release of titanium ions to the surrounding tissues [31,32] may be due to variable acidity, bacteria [33] and mechanical forces. These ions have the ability to generate some chronic inflammation and may also contribute to the development of peri-implantitis [34,35]. This complication is manifested by peri-implant soft tissue inflammation and also by progressive bone reabsorption [36]. Infection has the ability to damage the tissues surrounding the dental implant, automatically leading to implant loss [37]. Osseointegration is considered to be the most important step in the placement of a titanium dental implant [38]. Incomplete realization is considered to be another problem of using titanium in oral implantology. Even though specialists in this field appreciate this material for its high ability to bond with bone [39], this process is not guaranteed in all cases. There are a number of factors that can negatively influence the osseointegration process. Among them, we mention the patient’s bone quality, the existence of systemic diseases and the surgical technique. The occurrence of problems in the osseointegration process can lead to the instability of the dental implant and the need to remove it [40].

Another adverse effect of the use of titanium in oral implant dentistry is due to aesthetics and gingival transparency. Due to the colour of this metal, implants can become visible in the gingiva over time [41], an aspect especially identified in patients with very thin gums. This can lead to an unsightly result, especially when dental implants are anterior-placed. Furthermore, titanium dental implants can cause the discolouration of surrounding tissues [42]. To solve these problems related to the aesthetics of titanium dental implants, specialists have tried to use this material together with zirconia, forming the implant body from zirconia and the surface coating from titanium oxide. All this was carried out with the aim of solving the problems caused by the way the implants are visualized in the jaw [43]. In addition to these effects that can be identified in titanium dental implants, it should be mentioned that various fractures (in different parts of the implant) can occur [44], as well as mechanical failures that can influence its stability. Mechanical failures can be due to several factors, including design flaws or improper placement. These can influence both patient aesthetics as well as patient comfort [45].

Although titanium has certain effects on the human body, in recent years, there has been a significant increase in the number of patients choosing titanium dental implants. They are considered by patients and doctors to be the most durable and best fitting in the oral cavity. Over time, other types of dental implants, made of different materials, have emerged. Studies have shown that they are less toxic and adverse effects are almost non-existent compared to those made of titanium. There are currently no studies in the literature that analyse in detail patients’ perceptions of titanium dental implants, the advantages/disadvantages they offer and the risks perceived by patients regarding these medical devices. Furthermore, there are a limited number of studies investigating patients’ awareness of the adverse effects that may occur following the placement of a titanium dental implant.

In light of these observations, we determined it essential to conduct a study aimed at assessing patients’ perceptions of titanium dental implants, examining both the perceived advantages and disadvantages, as well as their awareness of potential adverse effects associated with these medical devices.

The objectives of this study centre on examining the factors that shape patients’ perceptions of titanium dental implants. Specifically, this study aims to analyse how perceived advantages, perceived challenges, awareness of associated risks, and any adverse effects encountered by patients influence their overall perception of these medical devices. Furthermore, this study investigates how these perceptions impact patients’ intentions to undergo similar implant procedures in the future.

The hypotheses that were the basis of the quantitative study are as follows:

**H1.**  *Respondents’ perceived advantages of titanium dental implants directly and positively influence their perception of these medical devices;*

**H2.**  *Problems perceived by respondents regarding titanium dental implants directly and negatively influence their perception of these medical devices;*

**H3.**  *The degree of awareness of the risks of titanium dental implants directly and negatively influences their perception of these medical devices;*

**H4.**  *The adverse effects experienced by respondents regarding titanium dental implants directly and negatively influence their perception of these medical devices;*

**H5.**  *Respondents’ perception of titanium dental implants directly and positively influences their intention to have a similar procedure in the future;*

**H6.**  *Respondents’ perceived advantages of titanium dental implants directly and positively influence their intention to use a new dental implant in the next 5 years;*

**H7.**  *Respondents’ perceived problems with titanium dental implants directly and negatively influence their intention to use a new dental implant in the next 5 years;*

**H8.**  *The degree of knowledge about the risks of titanium dental implants directly and negatively influences their intention to use a new dental implant in the next 5 years;*

**H9.**  *Adverse effects experienced by respondents regarding titanium dental implants directly and negatively influence their intention to use a new dental implant in the next 5 years.*

## 2. Materials and Methods

### 2.1. Conceptual Model

**Figure 1:**
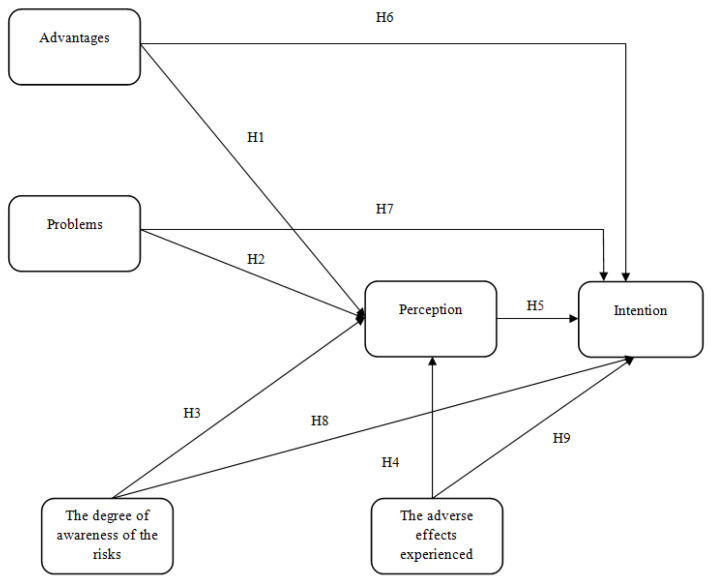
The proposed conceptual model.

Based on the hypotheses presented above, the following conceptual model (Figure 1) was drafted. Several hypotheses have been established to be tested.

### 2.2. Conducting the Research

Regarding the way the research was carried out, it should be mentioned that the quantitative study was conducted from August 2023 to June 2024. Thus, the results obtained are topical, illustrating the patients’ opinion regarding titanium dental implants as well as the adverse effects that may occur from their use.

The research group is identified with the survey unit. It is represented by patients who currently have at least one titanium dental implant. This research was conducted on a group of 162 respondents.

The following formula was used to make calculations for the group [46]:

$$N=\frac{t^{2}\times p\times (1-p)}{\Delta \omega^{2}}$$

*N* = the size of the group.

*t* = the coefficient associated with the probability of guaranteeing outcomes.

*p* = the proportion of patients participating in the study who have the researched characteristic.

Δω= the margin of error.

Using a 95% confidence level of the results, the *t*-value is 1.96. The margin of error considered was ±5% and the *p*-value was 0.5. Using these coefficients in the above formula, a group of 385 patients was obtained. Given the limited material and financial resources, the research was conducted on a group of 162 respondents.

In terms of how the research was carried out, it should be mentioned that before the final questionnaire was distributed to the respondents, it was tested on a number of 10 patients. After pre-testing, a number of amendments were made to the questionnaire, which was then posted on Google Forms. After that, the generated link was forwarded to several dental practises in Romania with the request to be given to patients who currently have a titanium dental implant. Respondents received by email the link where the questionnaire was posted and were given the opportunity to fill it in. In addition, they were asked if they knew other people who had a titanium dental implant in order to forward the questionnaire to them. The research method chosen was the survey, while the sampling method chosen was the snowball method. After finalizing the data collection procedure, the questionnaire was closed, and the database was downloaded and cleaned (incomplete answers and respondents who did not pass the filter question were removed). Subsequently, data analysis was performed using the IBM SPSS Statistics 28 programme.

### 2.3. Questionnaire Design

As regards the way the research instrument was developed, it should be mentioned that it was created taking into account the objectives and hypotheses that were established at the research level. At the beginning of the questionnaire, there was a filter question aimed at selecting the patients who were part of the researched group. Regarding the way in which the questionnaire was carried out, it should be pointed out that it was based on the following questions:

- Filter question: this question was designed to select only those persons who were part of the research population, i.e., patients who had at least one titanium dental implant to date.
- Descriptive questions: these were intended to determine the number of implants held by the respondents, how they were placed, the main reasons for choosing a titanium dental implant, the sources of information used in choosing a titanium dental implant, how the dental implant was chosen, the main benefits of these medical devices, and the main problems identified by the respondents in their choice of dental implant.
- Questions that addressed the hypotheses of the proposed conceptual model: these regarded perceived benefits, perceived problems, the awareness of the risks of titanium dental implants, adverse effects experienced by the respondents following the use of a titanium dental implant, respondents’ perception of titanium dental implants, and respondents’ intention to have a new titanium dental implant in the next period.
- Demographic questions were asked, aimed at identifying the respondents’ gender, age, last school graduated, labour market status, income and place of residence.

Regarding the types of scales used in this survey, the nominal scale, the interval scale, and the 7-step Likert scale (1—strongly disagree–7—strongly agree) were used to analyse the questions in the conceptual model.

The realization of the final questionnaire was carried out gradually. Several stages were thus completed. First, there was a pre-testing phase with 10 patients. The initial questionnaire was posted on an online platform and then sent to respondents. After this pre-testing stage, the research instrument was modified and some questions were corrected as some errors of wording and comprehension were identified. Then, after the testing phase, the questionnaire was posted on an online platform and distributed to patients. With regard to the sources of information that were used to carry out this research, it should be mentioned that data from secondary sources (previously conducted research, practical studies, analysis, clinical trials, etc.) as well as data from primary sources, obtained with the help of the questionnaire that was conducted at the level of this quantitative study, were used.

The reliability of the group was assessed using Cronbach’s alpha coefficient, which yielded a value of 0.732 > 0.7. This indicates the viability of the variables considered in the model.

## 3. Results

In terms of the results that were obtained from this study, it should be mentioned that 162 of the patients who participated in this research indicated that they currently have at least one titanium dental implant. Looking in terms of the number of titanium dental implants held by the patients at the moment, 39.5% of them have one dental implant, 23.5% of them have two such medical devices, while 37% of them have three or more titanium dental implants. Regarding the length of time that respondents have had a titanium dental implant, 26.5% of them have had their first dental implant for less than a year, 22.8% of them have had it for 1–2 years, 23.5% of patients have had it for 2–3 years, while 27.2% of them have had such a medical device for more than 3 years. Moreover, 30% of the patients have had intermediate implants for less than one year, 36.7% of them have had such devices for 1–2 years, 2–3 years (20%) or more than 3 years (13.3%). As for their last titanium dental implant, they have had it for either less than 1 year (30.6%), 1–2 years (34.7%), 2–3 years (21.4%) or more than 3 years (13.3%).

From the perspective of titanium dental implant placement, Table 1 shows that most patients had their dental implants placed in the same clinic by the same doctor (92%). In total, 1.2% of them had their dental implants placed in the same clinic but by different doctors, while 6.8% indicated that their titanium dental implants were placed in different clinics by different doctors.

Looking at the reasons that led patients to choose a titanium dental implant, 69.1% of them indicated that its resistance over time was the main element that led them to make this choice. Furthermore, 64.8% of the respondents mentioned the high success rate of implants made of this material, 32.1% of them consider that this medical device does not affect their body, 11.7% of them said that the low price of titanium dental implants made them choose this option, while 21% of them considered that the quick post-operative sale was the main factor that influenced them to do so.

With regard to the sources of information that patients used to find out about titanium dental implants, most of them indicated that they talked to dentists (72.2%) or family and friends (10.5%). Moreover, 10.5% of them had studied various specialized journals when choosing such a medical device, while other patients mentioned that they had researched via various websites (5.6%) or social media (1.2%). Analysing in terms of how the decision to use a titanium dental implant was made, respondents mentioned that they made this decision together with their dentist (74.7%) or alone (21.6%). Only a small proportion made the decision with family (3.7%).

In terms of the main benefits of titanium dental implants, the respondents considered the following: the restoration of natural teeth (45.1%), better aesthetics of teeth (37.7%), solving dental problems for a good value for money (38.3%) and the high success rate of titanium dental implants (57.4%). Another aspect studied in this research concerns the way in which doctors have communicated to patients all the information about possible problems that may arise from a titanium dental implant. Moreover, 88.9% of those who participated in the study indicated that their treating dentist informed them about this, while 11.1% indicated that he or she did not provide them with any information.

Analysing from the perspective of patients’ knowledge about the main problems that can occur after a titanium dental implant, most of them mentioned problems related to titanium hypersensitivity (52.1%), the yellow colouring of nails (2.1%), infections (29.9%), peri-implantitis (27.1%), changes in the colour of the gums (24.3%) and the possibility of the corrosion of the titanium dental implant (10.4%). In terms of the intensity with which patients experienced the adverse effects of titanium dental implants, the average was 1.46, which means that the effects experienced were quite low. In terms of the main reasons why respondents would have a new titanium dental implant in the next 5 years, 85.8% of respondents indicated that they would do so to replace a natural tooth, while 14.2% of patients would do so to replace an older dental implant.

Analysing in terms of demographic variables, it should be mentioned that 46.3% of those who participated in the study were women, while 53.7% were men. Looking in terms of the age range of the respondents, 6.8% of the patients are between 26 and 35 years, 37% of them are between 36 and 45 years, 38.3% of them are between 46 and 55 years, 15.4% of the respondents are between 56 and 65 years, and 2.5% of them are over 65 years. In terms of the distribution according to the last school graduated by the respondents, 5.6% of them have graduated vocational school, 16.7% of them have graduated high school, 45.1% of the respondents have graduated university, 29% of the patients have completed their master’s studies, while 3.7% have completed doctoral studies.

Regarding the net income of the patients, 16.7% of them have a net income between RON 2501 and 3500, 40.7% of the patients have a monthly income between RON 3501 and 4500, and 42.6% of the respondents have more than RON 4500. Regarding the residence environment of the respondents, 90.1% of them live in urban areas, while 9.9% of them live in rural areas. Looking at the labour market status of the patients, 15.4% of them are directors/managers or have managerial positions, 13% of them are employers or administrators, 37.7% are specialists with a university degree, 1.2% of them are civil servants that gradated high school, 12.3% of the patients are employed in trade and services, 11.1% are skilled workers, 3.1% are self-employed, 1.2% of them are pupils/students and 4.9% are retired.

### SEM Analysis Using WarpPLS 8.0

**Figure 2:**
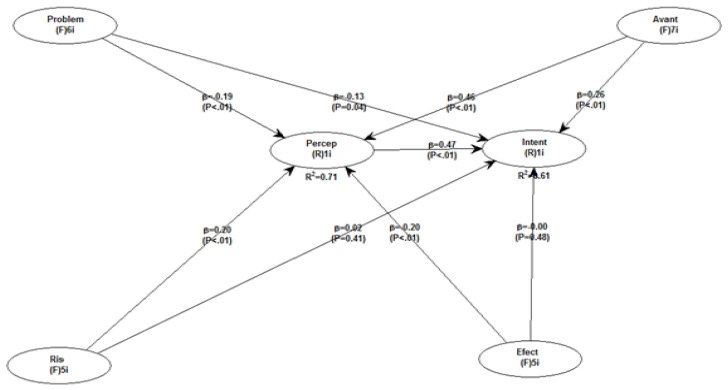
Pattern of respondents’ perception of titanium dental implants.

WarpPLS 8.0 was used to test the model proposed in this paper. Its role was to analyse the independent variables that have the ability to influence patients’ perception of titanium dental implants. The latent variables analysed in this econometric model were the following: perceived benefits, perceived problems, the perceived awareness of the risks of titanium dental implants, and perceived adverse effects of titanium dental implants (Figure 2).

In the figure above, the path coefficients are mentioned as “Beta coefficients”. The corresponding *p*-values are displayed within parentheses, while the R-squared values are presented beneath each endogenous latent variable. With regard to the R2 value, it should be mentioned that it shows the extent to which the variation in the dependent variable is explained by the independent variables that have been taken into account in the model. According to the results obtained, 71% of the patients’ perception of titanium dental implants is explained by the independent variables that are analysed at the level of the model, while for 61%, their intention to have a new titanium dental implant in the next period is explained by these independent variables.

The hypotheses established in this research aimed at identifying the link between several variables established in the proposed conceptual model. Table 2 shows the main hypotheses initially established as well as the β coefficients obtained after testing this model. The obtained results illustrated that seven hypotheses out of nine were accepted, while two of them were rejected. Hypothesis 8 and Hypothesis 9 were rejected because the significance limit *p*-value was greater than 0.05 (in the case of Hypothesis 8, it registered a value of 0.41, and in the case of Hypothesis 9, it registered a value of 0.48).

In order to validate a particular model, several indicators of compliance need to be analysed. In Table 3, it can be observed the results that were obtained after testing it in WarpPls. The value of *p* was less than 0.001 for the indicators analysed in this econometric model. Moreover, the value of the APC was 0.215, that of the ARS indicator was 0.661 and that of the AARS was 0.651. The value of the AVIF indicator was 1.972, a value lower than 3 which illustrates an ideal situation for the proposed model. AFVIF had a value of 2.271, less than 3, which is a very good value for the model. The value of GoF is 0.744, which illustrates that the proposed model is accepted. SPR was 0.9, and the RSCR, SSR, and NLBCDR have the value of 1, confirming that the proposed conceptual model is accepted.

## 4. Discussion

Titanium (Ti) is considered to be a silvery-coloured metal that has high strength over time but low density. Looking from the point of view of the most important property of this material, it should be stated that it is given by its high chemical stability, which makes it more resistant in terms of corrosion [47]. Ever since its use in oral implant dentistry, titanium dental implants have been seen as the best option in terms of restoring dento-maxillary functions through the process of replacing natural teeth that have been lost or damaged [48]. Currently, most dentists turn to dental implants made of titanium or titanium alloys [49], considering that they have high biocompatibility, present a tight bone bond and have high resistance to wear and fracture [50]. Although titanium and titanium alloys are materials used in dental implantology due to their remarkable properties, specialists in this field have identified that there is a possibility of certain adverse effects [51].

In addition to these, it has been observed that patients who receive a titanium dental implant may experience allergic reactions [52], titanium hypersensitivity [53], peri-implantitis [54], corrosion [55], incomplete osseointegration or aesthetic problems. These are just some of the issues to be considered by both dentists and patients. Given that there is currently a high risk of failure in these types of dental implants, dentists very often administer antibiotics as a preventive measure [56]. Studies in this field have illustrated that administering 2–3 g of amoxicillin within 1 h of surgery, followed by 500 mg/8 h for 5–7 days, reduces the rate of the early failure of these medical devices. According to the results of several studies, azithromycin, clarithromycin or metronidazole are recommended for patients who are allergic to penicillin [57]. On the other hand, in the case of titanium allergies, the dentists can perform Melisa tests (memory lymphocyte immunostimulation assay) [24]. These tests help doctors to identify patients with metal allergies. Melisa is a blood test that illustrates patients’ sensitivity to various metals, including titanium. One of the issues that has been emphasized in recent years is the possibility of titanium particles being released after placement. Studies [58] in this field have illustrated that titanium particles can be identified around the soft tissues in the vicinity of the screw in the medical device after the fabrication of a titanium dental implant. Thus, continued research in this field and new materials and disruptive technologies [59] on the market have the potential to undoubtedly contribute to improved outcomes and reduced risks associated with titanium dental implants.

An important aspect that must be pointed out about dental implants refers to the fact that, from the time of their appearance until today, several materials have been used in their realization. Titanium was not the first material from which they were made. Thus, dental implants have been made of [60] dental metals and alloys (unalloyed titanium (cpTi), Tital alloys (Ti-6AI-4V, Ti-6AI-7Nb, Ti-5Al-2. 5Fe), gold alloys, non-noble alloys (Co-Cr-Mo, Fe-Cr-Ni, stainless steel as well as Tantalum), ceramics (this category includes those made of Al oxide, Ti oxide, Zr oxide, bioactive and biodegradable ceramics, β-Tricalcium phosphate, vitreous carbon, bio glass, and carbon–silicon) and those made of polymers and composites. Even though dental implants made of other metals have a number of biomechanical properties, they have gradually been replaced by titanium and its alloys because the latter have a much higher clinical success rate compared to those made of other metals. However, the problems identified with titanium dental implants have encouraged specialists to identify other materials from which to manufacture these medical devices so that the adverse effects on the human body are minimal or non-existent. Technological developments in recent years have led to a careful consideration of how ceramics can be used in this field [61].

As an alternative to dental implants made of titanium [62], those made of zirconia [63,64] have been approved on the European market for more than 20 years. In recent years, the market for zirconia dental implants has grown tremendously, with specialists estimating that it will grow from USD 4.99 billion globally in 2023 to USD 9.62 billion by 20,230 [65]. At this time, there are a number of differences between dental implants made of titanium and those made of zirconia. From the point of view of how titanium dental implants are made, they consist of two parts (the implant body and the abutment), while zirconia dental implants are made of one piece [66]. From a cost perspective, zirconia dental implants have a higher cost compared to titanium dental implants, and from the aesthetic point of view, it should be mentioned that zirconia dental implants have much better aesthetics [67]. Previous studies on patients’ perception of titanium dental implants [68] illustrated that patients were satisfied overall with these medical devices regarding their strength and stability but preferred zirconia dental implants for the soft tissue colour.

In another study, at the level of the specialty literature [69], it was observed that titanium dental implants offer a number of advantages to patients, the most important of which are functional improvement and increased social confidence. The main disadvantages mentioned by patients were their high cost, previous experience with another dental implant and the duration of treatment. Another research study conducted in this area [70] illustrated that the experience that patients had following a dental implant was a positive one, even though they indicated that prior to the procedure, they did not have much knowledge about the materials that can be used, the adverse effects that can occur after their placement or the procedures associated with them. Because of this, it was noticed that there was a need to educate patients about this medical procedure by dentists.

Thus, they should discuss the treatment openly with patients from the very beginning, explain why a dental implant is necessary, present the alternatives available on the market (the materials they are made of), the advantages and disadvantages of each of them and the procedures available in their case. Thus, following such a discussion, patients would better understand the benefits they can gain from having a dental implant of a certain type placed as well as the risks they are exposed to and thus be able to make a decision based on clear, accurate and factual information received from a specialist. Analysing in terms of the main means of information that patients currently use to inform themselves about dental implants, specialists [71] identified the following: patient information panels, printed advertisements, messages posted by individual patients or companies in the field on social networks, and patients’ personal connections. However, all the details that patients find in these promotional materials only provide information about the main benefits or costs, but they do not provide details about possible adverse reactions that may occur later. In this study, patients indicated that their main reasons for using titanium dental implants are to restore their physical appearance and to increase their quality of life.

Another study in this field [72] aimed to identify patient satisfaction with titanium dental implants 15 years after an implant procedure. The results illustrated that the respondents were very satisfied with their survival rate (93% of the medical devices survived 10 years), the chewing comfort (more than 97% of them), and their phonetic function and aesthetics (97% of the study participants). A third of respondents said that titanium dental implants are much easier to clean than natural teeth. However, about 50% of those who took part in the study experienced bleeding gums after brushing, with more bleeding around implants than around other teeth. This study illustrated that 94% of patients who have had a titanium dental implant intend to have a titanium dental implant in the near future and would recommend dental implants to friends, relatives or close friends.

The study conducted in this paper aimed to identify patients’ perception of titanium dental implants and their adverse effects on the human body. The data analysis showed that 74.7% of the patients took the decision to have a titanium dental implant together with their dentist. The conceptual model proposed in this paper aimed to identify how a series of independent variables (perceived advantages, perceived problems, the degree of knowledge of the existing risks regarding titanium dental implants, and adverse effects felt by the respondents following the use of a titanium dental implant) have the ability to influence both the patients’ perception of titanium dental implants and their intention to have a new dental implant in the next period. The proposed econometric model was validated, with seven hypotheses accepted out of nine.

In terms of the limitations of this research, it should be mentioned that several limitations were identified in this study, both in terms of the way the research was conducted and in terms of the way the results were collected and interpreted. The first limitation concerns the small number of respondents who participated in the research (162). This does not make it possible to extrapolate the results to the entire research community. In order to have an overview of the subject under study, further studies should be carried out in the future with larger sample sizes and with greater clarity on the adverse effects experienced by the respondents following the use of a titanium dental implant.

Still from the respondents’ point of view, it should be mentioned that another limitation of the research concerns their self-selection. Thus, it is possible that those who chose to participate in this online survey have a higher interest or inclination towards oral hygiene. In addition, it is likely that more people who had positive/negative experiences with titanium dental implants participated, and because of this, they were willing to share their opinion on the topic. Another limitation of this study relates to response bias. With this being a study conducted in the field of oral implant dentistry, it is possible that the patients who participated in this research may have been influenced by a number of factors when answering the questions. Thus, it is possible that a number of factors such as how they experienced or underestimated pain, their satisfaction with a titanium dental implant and their willingness to respond politely to the survey may have influenced the way they answered the questions.

Another limitation of this research concerns the access and availability of respondents. With it being an online survey, it is possible that only those who have access to technology and those who are knowledgeable in completing an online survey responded. Those who do not have access to such technology or mobile devices were unable to complete the survey, and thus, the accuracy and representativeness of the data were affected. In terms of the variables that were considered in this research, it should be mentioned that only certain variables that were considered to be important in the field under study were used. It is possible that in addition to these, there are others which have not been analysed. For this reason, in future studies, it is very important to take into account other variables that have been previously studied in the literature. Furthermore, a series of qualitative studies (focus groups, in-depth interviews, etc.) should also be carried out among doctors in order to observe their opinion on the main symptoms experienced by their patients following the placement of a titanium dental implant.

Given the results obtained in this research, it should be mentioned that future studies should analyse this topic in detail and identify both the perception of dentists regarding titanium dental implants and the adverse reactions they have encountered over time. Thus, in the next period, it is necessary to conduct qualitative research (focus groups, in-depth interviews, etc.) in order to observe the opinion of dentists on this topic and their attitudes towards implants made of other materials. In addition, the extent to which medical professionals are able to influence patients’ decisions and their willingness to work with dental implants made of other materials (which do not have such adverse effects) in the future should also be studied.

In addition to these qualitative studies, other research studies of a quantitative nature should be carried out among both patients and dentists. Those among dental professionals should study their opinion on titanium dental implants. This could be carried out by comparing doctors working in the public sector versus those working in the private healthcare system. In addition, significant differences should be identified between doctors in terms of their experience, the city in which they work and their openness to new technologies (ex. AI, the Internet of Things, cloud technology, augmented reality, virtual reality, etc.) [73] in the field. Further research among patients should build on the results of this study. In addition, other variables that for some reason have not been analysed so far should be taken into account. These studies should examine significant differences between patients in terms of region of residence, net income and education level.

## 5. Conclusions

Titanium is a highly valued material in dental implantology due to its exceptional properties of biocompatibility, strength and durability. Although this material is used to a very high extent in this field, its application also has some adverse effects. Studies carried out in this sector in recent years have illustrated that its application can lead to hypersensitivity to titanium, inflammation, allergies, pain or irritation. In order to minimize the adverse effects of this material on the human body, it is very important that patients are continuously monitored by their doctors in order to quickly detect and manage any adverse effects that may occur, thus ensuring the long-term success of treatment with titanium dental implants.

The results of this study illustrated an overview of patient perceptions of titanium dental implants. In addition, they illustrated the main reasons that led patients to choose such a medical device as well as the influence that dentists have on them in the decision-making process. This research provides valuable information both to doctors working in the field and to patients who are considering a dental implant in the near future. It illustrates the main factors that have the potential to influence both patients’ perception of titanium dental implants and their intention to use such medical devices in the near future.

## Figures and Tables

**Table 1:** Titanium dental implant placement.

Criteria		Frequency (%)
Titanium dental implant placement		
	The placement of titanium dental implants in the same clinic by the same doctor	92%
	The placement of titanium dental implants in the same clinic but by different doctors	1.2%
	The placement of titanium dental implants in different clinics and by different doctors	6.8%
	Total	100%
	Information sources used by respondents	
	Dentists	72.2%
	Specialty magazines	10.5%
	Family and friends	10.5%
	Websites	5.6%
	Social networks	1.2%
	Total	100%
	Decision to use titanium dental implants	
	Alone	21.6%
	Together with your family	3.7%
	With the dentist	74.7%
	Total	100%

Source: statistical survey made by the authors.

**Table 2:** Validation of hypotheses using the variation method.

No.	Hypothesis	β	*p*	Validation
H1	Perceived benefits—perception	0.46	<0.01	Yes
H2	Perceived problems—perception	−0.19	<0.01	Yes
H3	Awareness of existing risks—perception	0.20	<0.01	Yes
H4	Adverse effects experienced—perception	0.20	<0.01	Yes
H5	Perception–intention	0.47	<0.01	Yes
H6	Perceived benefits—intention	0.26	<0.01	Yes
H7	Perceived problems—intention	−0.13	=0.04	Yes
H8	Awareness of existing risks—intention	0.02	=0.41	No
H9	Adverse effects experienced—intention	−0.00	=0.48	No

Source: statistical survey made by the authors.

**Table 3:** Coefficients measuring the compliance of the research model.

Indicators	Validation Criteria
Average path coefficient (APC) = 0.215	*p* < 0.001
Average R-squared (ARS) = 0.661	*p* < 0.001
Average adjusted R-squared (AARS) = 0.651	*p* < 0.001
Average block VIF (AVIF) = 1.972	Accepted if value obtained is ≤5, ideal ≤3.3
Average full collinearity VIF (AFVIF) = 2.271	Accepted if value obtained is ≤5, ideal ≤3.3
TenenhausGoF (GoF) = 0.744	Accepted if obtained value is low ≥0.1, medium ≥0.25, high ≥0.36
Sympson’s paradox ratio (SPR) = 0.9	Accepted if value obtained is ≥0.7, ideal = 1
R-squared contribution ratio (RSCR) = 1.000	Accepted if the obtained value is ≥0.9, ideal = 1
Statistical suppression ratio (SSR) = 1.000	Accepted if obtained value is ≥0.7
Nonlinear bivariate causality direction ratio (NLBCDR) = 1.000	Accepted if value obtained is ≥0.7

Source: statistical survey made by the authors.

## Data Availability

The original contributions presented in this study are included in the article, and further inquiries can be directed to the corresponding author.
